# A randomised controlled trial of calcium channel blockade (CCB) with Amlodipine For the treatment oF subcortical ischaEmic vasCular demenTia (AFFECT): study protocol

**DOI:** 10.1186/s13063-016-1449-3

**Published:** 2016-07-18

**Authors:** Caroline Greenan, Lynn Murphy, Ly-Mee Yu, Patrick G. Kehoe, Elizabeth Coulthard, Philip Bath, Robert Stewart, Rob Jones, Anne Corbett, Alan Thomas, Peter Connelly, Frank Arrojo, Rachel Canning, Sylvia Wallach, Catherine Henderson, Bernadette McGuinness, Mike O’Sullivan, Clive Holmes, Martin Knapp, Clive Ballard, Peter Passmore, AFFECT Investigators, Peter Passmore, Eric Jackson, Roy Soiza, Peter Connelly, Rob G. Jones, Vanessa Raymont, Latha Velayudhan, Alan Thomas, Rohan Vanderputt, Stephen Pearson, Robert Lawrence, Clive Ballard, Frances Harrington

**Affiliations:** Northern Ireland Clinical Trials Unit, 1st Floor Elliott Dynes Building, Royal Victoria Hospital, Grosvenor Road, Belfast, BT12 6BA UK; Dementia Research Group, Clinical Neurosciences, University of Bristol, Level 1 Learning and Research Building, Bristol, BS10 5NB UK; Clinical Sciences Building, Nottingham City Hospital, Hucknall Road, Nottingham, NG5 1PB UK; Institute of Psychiatry, Psychology & Neuroscience, King’s College London, 16 De Crespigny Park, London, SE5 8AF UK; School of Community Health Sciences, Division of Psychiatry, Institute of Mental Health, University of Nottingham, Innovation Park, Triumph Road, Nottingham, NG7 2TU UK; Wolfson CARD, Kings College London, Wolfson Wing, Hodgkin Building, Guy’s Campus, London, SE1 1UL UK; Biomedical Research Building, Institute of Neuroscience and Newcastle University Institute for Ageing, Newcastle University, Campus for Ageing and Vitality, Newcastle upon Tyne, NE4 5PL UK; Murray Royal Hospital, Muirhall Road, Perth, PH2 7BH UK; Research Network Volunteer, Alzheimer’s Society, 58 St Katharine’s Way, London, E1W 1LB UK; Primary Care Clinical Trials Unit, Nuffield Department of Primary Care Health Sciences (Gibson Building), Radcliffe Observatory Quarter, Woodstock Road, Oxford, OX2 6GG UK; Personal Social Services Research Unit (PSSRU), London School of Economics, Houghton Street, London, WC2A 2AE UK; Centre for Public Health, Institute of Clinical Sciences, Block B, Queen’s University Belfast, The Royal Hospitals, Grosvenor Road, Belfast, BT12 6BA UK; Academic Neuroscience Centre, Institute of Psychiatry, De Crespigny Park, PO Box 41, London, SE5 8AF UK; MARC, University of Southampton, Moorgreen Hospital, Botley Road, Southampton, S030 3JB UK; Wolfson Centre for Age-Related Diseases, King’s College London, Guy’s Campus, London, SE1 1UL UK

**Keywords:** Vascular dementia, Subcortical ischaemic vascular dementia, Amlodipine, Calcium channel blockade, Cognitive outcome

## Abstract

**Background:**

Vascular dementia is the second most common cause of dementia affecting over seven million people worldwide, yet there are no licensed treatments. There is an urgent need for a clinical trial in this patient group. Subcortical ischaemic vascular dementia is the most common variant of vascular dementia. This randomised trial will investigate whether use of calcium channel blockade with amlodipine, a commonly used agent, can provide the first evidence-based pharmacological treatment for subcortical ischaemic vascular dementia.

**Methods/Design:**

This is a randomised controlled trial of calcium channel blockade with Amlodipine For the treatment oF subcortical ischaEmic vasCular demenTia (AFFECT) to test the hypothesis that treatment with amlodipine can improve outcomes for these patients in a phase IIb, multi-centre, double-blind, placebo-controlled randomised trial.

The primary outcome is the change from baseline to 12 months in the Vascular Dementia Assessment Scale cognitive subscale (VADAS-cog). Secondary outcomes include cognitive function, executive function, clinical global impression of change, change in blood pressure, quantitative evaluation of lesion accrual based on magnetic resonance imaging (MRI), health-related quality of life, activities of daily living, non-cognitive dementia symptoms, care-giver burden and care-giver health-related quality of life, cost-effectiveness and institutionalisation.

A total of 588 patients will be randomised in a 1:1 ratio to either amlodipine or placebo, recruited from sites across the UK and enrolled in the trial for 104 weeks.

**Discussion:**

There are no treatments licensed for vascular dementia. The most common subtype is subcortical ischaemic vascular dementia (SIVD). This study is designed to investigate whether amlodipine can produce benefits compared to placebo in established SIVD. It is estimated that the numbers of people with VaD and SIVD will increase globally in the future and the results of this study should inform important treatment decisions.

**Trial registration:**

Current Controlled Trials ISRCTN31208535. Registered on 7 March 2014.

## Background

Worldwide there are approximately 35.6 million people with dementia and this is expected to rise significantly in the next decade [1].Vascular dementia (VaD) is the second most common cause of dementia (approximately 20 %) accounting for over seven million people worldwide. It is estimated that VaD affects 1–4 of every 100 individuals aged 65 years [2] and the prevalence increases to 14–16 of every 100 people over 80 years of age [3].

VaD can arise due to a number of different underlying cerebrovascular pathologies that were defined according to a consensus classification of Vascular Cognitive Impairment developed by international experts under the auspices of the International Psychogeriatric Association [4]. VaD is very heterogeneous condition that has been a major barrier to developing and evaluating effective therapies.

Subcortical ischaemic vascular dementia (SIVD) is the most common form of VaD and results from small-vessel disease. This disease produces either cavitating lesions in white or subcortical grey matter called lacunes and diffuse damage to white matter connections often attributed to incomplete infarction due to critical stenosis of medullary arterioles and hypoperfusion. Symptoms include motor and cognitive slowing, difficulty with complex tasks (executive dysfunction), problems with memory retrieval, dysarthria, mood changes, urinary symptoms, and short-stepped gait [5]. These manifestations probably result from ischaemic interruption of parallel circuits from the prefrontal cortex to the basal ganglia and corresponding thalamocortical connections. Brain imaging [computed tomography (CT) and magnetic resonance magnetic resonance imaging (MRI)] has been instrumental in revealing the prevalence of subcortical lesions, rejuvenating the concept of SIVD, and provides a ready means to identify subcortical vascular disease, which is needed to reach a diagnosis of SIVD in practice.

SIVD is a priority candidate for development and evaluation of effective treatments for VaD for a number of reasons. First, SIVD is the most common form of VaD and there is a need to focus on people with more homogeneous forms of VaD to effectively develop and evaluate new therapies that could realistically be implemented in clinical practice. In addition, the strong age-correlated prevalence of subcortical lesions combined with an ageing population means that the prevalence of SIVD is increasing. Alongside the age-related rise in subcortical pathology, the relative contribution of SIVD to vascular dementia is rising. Furthermore, progress in the treatment of large-vessel vascular disease has unfortunately not been accompanied by improvements in treatment of small-vessel disease, including SIVD [6].

A Cochrane review identified 15 studies examining the benefit conferred by calcium channel blockers (CCBs) in dementia, including Alzheimer’s disease (AD), VaD and non-specific dementia types. Of these studies ten specifically examined VaD [7], the majority of which were very small and did not use operationalised diagnostic criteria to recruit participants. However, the review highlighted three studies that involved more than 50 people with VaD according to operationalised criteria. Of these, two studies of 12 weeks duration included 67 and 62 participants respectively, and showed benefits in cognitive function and global clinical outcome [8, 9]. The largest reported study, which included 259 people over 6 months found no significant benefit in cognitive outcomes in the overall study population of people with VaD [10]. However, a post hoc subgroup analysis of 92 people with SIVD from this study showed significant improvement in both cognitive and functional outcome measures, contrary to the lack of effect seen in a subgroup of people with multi-infarct dementia. The authors highlighted the need for a larger a priori trial of CCBs specifically in people with SIVD [10].

### Study rationale

VaD is a highly significant health issue, affecting millions of people worldwide. This condition presents a substantial challenge and burden for health service provision, health economics and both informal and formal care. Despite this there are no effective pharmacological treatments. Developing new, effective treatments is therefore an urgent imperative to ensure this enormous unmet need is addressed.

SIVD is a progressive condition with many patients showing hypertension and hypercholesterolaemia, in addition to other cardiovascular comorbidities. Some studies have been conducted and further ongoing studies are evaluating the prevention of post-stroke dementia. However, to date, AFFECT is the only registered pharmacological treatment trial for SIVD.

CCBs have a consistent effect on stroke reduction [11–14], can alter calcium flux in a beneficial way in neurons [15] and have been shown to improve memory in hypertensive patients [16]. The previous studies in VaD and in SIVD in particular, strongly suggest the need for a properly designed randomised clinical trial to investigate CCBs in SIVD.

Amlodipine is a commonly used CCB that is now available generically at doses of 5 mg and 10 mg. It is usually initiated at the 5 mg daily dose and titrated to 10 mg. In this study, the maximum tolerated dose will be used, as this is most likely to demonstrate an effect without the compromise of side effects.

## Methods/Design

The Belfast Health and Social Care Trust (BHSCT) is the Sponsor for this trial that will be conducted in accordance with the ethical principles that have their origin in the Declaration of Helsinki. The protocol was approved by the Office for Research Ethics Committees, Northern Ireland (14/NI/0069). The trial is registered on the International Standard Randomized Controlled Trial Registry (ISRCTN31208535ISRCTN31208535) and with the European Union Drug Regulating Authorities Clinical Trials database (2014-000926-39). The study is funded jointly by the Alzheimer’s Society and British Heart Foundation and is being coordinated by the Northern Ireland Clinical Trials Unit (NICTU) (http://www.nictu.hscni.net). The trial will comply with the principles of good clinical practice (GCP) and will be carried out in accordance with applicable legislation and the standard operating procedures of the NICTU. The trial will be reported in line with the Consolidated Standards of Reporting Trials (CONSORT) 2010 guidelines.

### Study design

The AFFECT trial is a multi-centre, randomised, double-blind, placebo-controlled, parallel phase IIb trial of amlodipine in patients with SIVD.

**Fig. 1:**
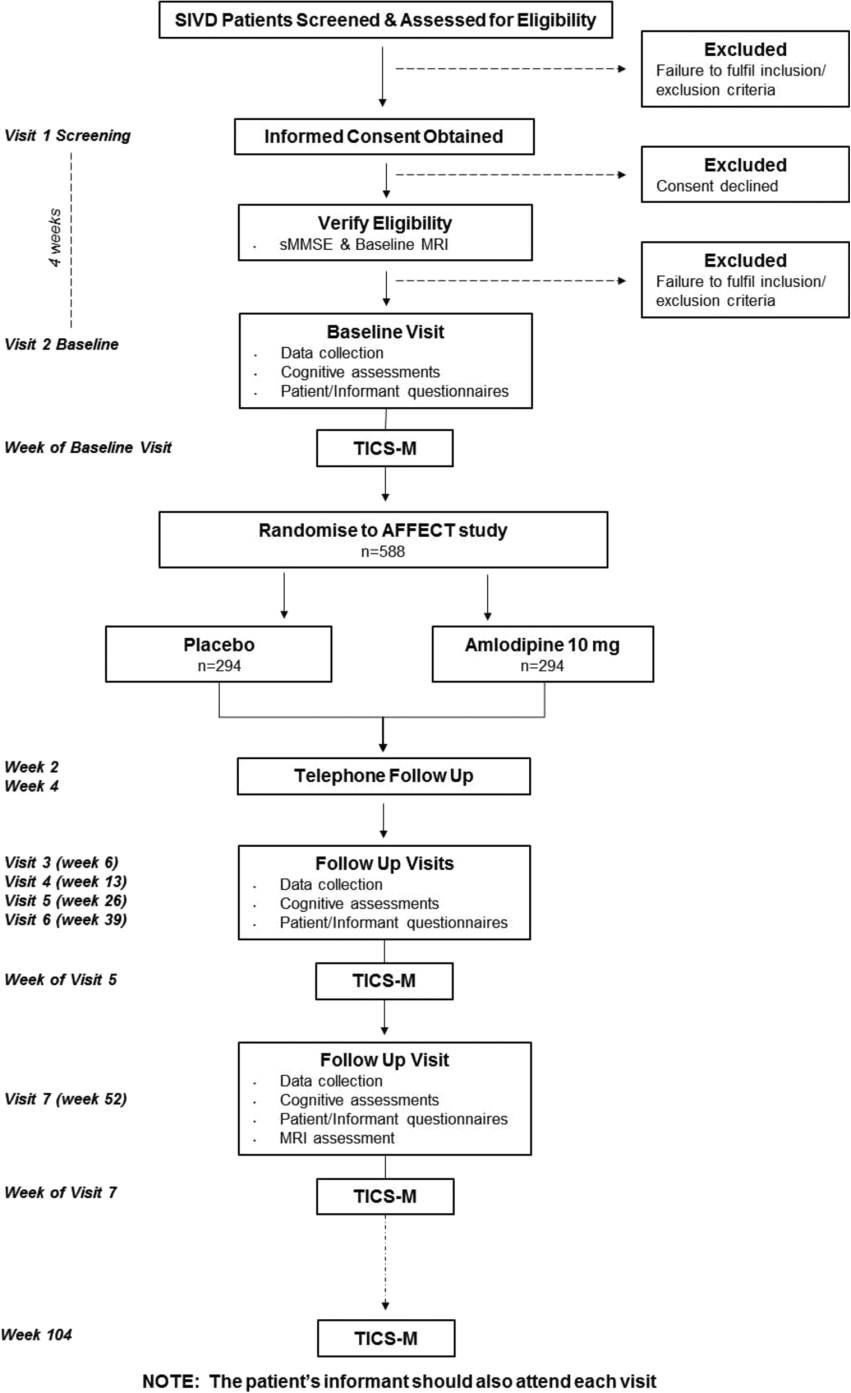
Study schematic for the AFFECT trial

Patients with SIVD fulfilling the eligibility criteria will be randomised in a 1:1 ratio to either amlodipine or matching placebo: 5 mg once daily for 2 weeks followed by 10 mg once daily for 50 weeks. A total of 588 patients aged above 50 years from a community setting will be recruited from sites across the UK. Patients will be enrolled in the study for 104 weeks; 52 weeks on treatment and a follow-up telephone call at 104 weeks. Patients will attend the clinic for seven visits throughout the study at screening, baseline, 6, 13, 26, 39, 52 weeks as outlined in Fig. 1.

### Outcome measures

The primary outcome measure will be a change from baseline to 12 months in Vascular Dementia Assessment Scale cognitive subscale (VADAS-cog) score. The VADAS-cog is a more detailed cognitive assessment designed to be more sensitive than the Alzheimer’s Disease Assessment Scale-Cognitive subscale (ADAS-Cog) to the cognitive outcomes in people with vascular dementia [17]. This assessment should be completed face to face by an assessor who will be blinded to the intervention.

There are a number of secondary outcomes for this trial that include:

- Change in cognitive function measured with the standardised Mini-Mental State Examination (sMMSE) from baseline to 12 months [18].
- Change in the Trail Making test B [19] from baseline to 12 months. The Trail Making test B is a timed measure of executive function.
- Change in cognitive function from baseline to 12 months measured by Modified Telephone Interview for Cognitive Status [20]. There will also be a follow-up at 24 months.
- Change in Clinical Global Impression of Change (CGIC) [21] from baseline to 12 months. CGIC is a simple standardised rating of overall clinical outcome, rated by a clinician blind to treatment allocation.
- Change in blood pressure from baseline to 12 months.
- Change in lesion accrual from baseline to 12 months. This will be based on quantitation of lacunar lesions and diffuse white matter lesions measured quantitatively by MRI.
- Change in patient-reported health-related quality of life from baseline to 12 months measured with the EuroQol Group EQ-5D Health Questionnaire (EQ-5D) [22] and the Dementia Quality of Life-Proxy [23], a carer-rated and disease-specific measure of quality of life in dementia.
- Change in activities of daily living (ADL) from baseline to 12 months measured using the Disability Assessment in Dementia [24].
- Change in non-cognitive dementia symptoms from baseline to 12 months measured with the Neuropsychiatric Inventory Caregiver Distress [25].
- Change in caregiver burden from baseline to 12 months measured with the 12-item General Health Questionnaire [26], and care-giver health-related quality of life measured with the EQ-5D-5 level (5L).
- Cost-effectiveness measured as the combination of costs generated from the Client Service Receipt Inventory [27] and effectiveness measured by VADAS-cog, quality-adjusted life-years (QALYs) from DEMQOL-Proxy and EQ-5D-5L.
- Institutionalisation defined as permanent transition from living in an independent household to a care home, nursing home, National Health Service (NHS) continuing care unit or hospital and measured with questions taken from the client service receipt inventory (CSRI).

### Eligibility criteria

Patients will be eligible to participate in the study if they fulfil all of the following inclusion criteria:

1. Dementia syndrome according to the criteria a, b and d from code 290.4 of the *Diagnostic and Statistical Manual of Mental Disorders, Fourth Edition* (DSM-IV) [28].
2. Evidence of one or more clinical features in support of SIVD such as executive dysfunction, mood or gait disturbance or focal neurological signs [29]. Patients who present solely with low mood and a small number of lacunar lesions will only be included if there are additional indicators from other domains (e.g. gait, neurological signs, executive dysfunction).
3. Multiple lacunae (>2) or diffuse lesions reaching a mean Fazekas score of 2–3 across brain regions identified on baseline MRI scan.
4. sMMSE score between 15 and 26 (inclusive).
5. Age ≥ 50
6. Evidence of adequate intellectual functioning so that patient is capable of giving consent.
7. In patients taking a cholinesterase inhibitor or memantine, dose stable for at least 3 months. All subjects taking cholinesterase inhibitors or memantine will have to be vetted for inclusion by the Diagnostic Monitoring and Event Adjudication (DMEA) Committee to ensure that the primary diagnosis is not AD.
8. In patients taking antidepressants, dose stable for at least 4 weeks.
9. CT or MRI scan consistent with the probable diagnosis of SIVD providing there has been no significant clinical change since the scan.
10. Patient has resident family or professional carer or is visited at least twice a week by carer.
11. Fluency in English is essential as the study requires questionnaires to be completed.
12. Likely to be able to participate in all scheduled evaluations and complete all required tests.
13. Provision of appropriate consent.
14. Presence of an informant, aged 18 years or over who is willing to participate in the study.

Patients fulfilling any of the criteria below will be excluded from the trial:

1. Severe, unstable or poorly controlled medical conditions apparent from physical examination or clinical history.
2. Moderate/severe heart disease or severe hepatic disease.
3. Significant renal insufficiency; estimated glomerular filtration rate (eGFR) <30 ml/min.
4. Blood pressure (sitting) exceeds 160 mmHg systolic and/or 110 mmHg diastolic.
5. Systolic blood pressure (sitting) is less than 110 mmHg.
6. Infarction involving the cortex on MRI scans.
7. Cerebrovascular event within the last 6 months.
8. Myocardial infarction within the last 3 months.
9. Already taking any calcium channel blocker.
10. Contraindications to a calcium channel blocker as per summary of product characteristics (SPC).
11. Patient is unable to take trial medications.
12. Pregnant women, women who have not yet reached the menopause (no menses for ≥12 months without an alternative medical cause) who test positive for pregnancy, are unwilling to take a pregnancy test prior to trial entry or are unwilling to undertake adequate precautions to prevent pregnancy for the duration of the trial.
13. Female patients who are breastfeeding.
14. AD is considered to be the primary diagnosis, i.e. a predominantly amnestic presentation or evidence of an amnestic (pre-dementia) phase or strong biomarker evidence to support a diagnosis of AD. Patients with severe hippocampal atrophy on MRI (Scheltens GR 3 and 4 on both sides [i.e. a total score (left plus right) of 6 or more will be excluded].
15. Significant neurological disease that may affect cognition other than SIVD or AD as a concurrent pathology.
16. Current presence of a clinically significant major psychiatric disorder (e.g. Major Depressive Disorder) according to the criteria of the DSM-IV.
17. Current clinically significant systemic illness that is likely to result in deterioration of the patient’s condition or affect the patient’s ability to complete the study or their safety during the study.
18. Treatment with immunosuppressive medications (e.g. systemic corticosteroids) within the last 90 days (topical and nasal corticosteroids and inhaled corticosteroids for asthma are permitted) or chemotherapeutic agents for malignancy within the last 3 years.
19. Other clinically significant abnormality on physical, neurological, laboratory, examination that could compromise the study or be significantly detrimental to the patient (e.g. postural hypotension diagnosed within the last year which in the opinion of the Principal Investigator (PI) would exclude the patient).
20. Alcohol or drug dependence or abuse within the last 2 years.
21. Treated with any other investigational medication or device within 60 days.
22. Patient taking simvastatin 40 mg or greater. A patient may be switched to an alternative statin and on stable dose for 3 months to meet the inclusion criteria. A reduction in simvastatin dose solely for the purposes of eligibility is not permitted.

## Trial conduct

### Withdrawal of consent

Patients may withdraw from the trial at any time without prejudice. Permission will be sought for members of the study team to access medical records for data related to the trial. Data recorded up to the point of withdrawal will be included in the analysis, unless consent to use their data has also been withdrawn.

Informants may withdraw from the trial at any time. An attempt will be made to recruit an alternative informant for the patient and consent obtained. However, if this is not possible the patient will continue to be followed up as part of the trial, unless the patient also withdraws from the trial.

If the patient and/or informant requests termination of the trial drug during the treatment period, the drug will be stopped but the patient will continue to be followed up as part of the trial, unless the patient withdraws from the trial.

### Randomisation procedure

Patients will be randomised with an allocation ratio of 1:1 amlodipine to placebo using Sortition, an online randomisation system developed by the Primary Care Clinical Trials Unit (PC-CTU) at the University of Oxford.

A non-deterministic minimisation algorithm will be used to ensure balanced allocation of patients across the two treatment groups for the following important prognostic factors: vascular risk score, smoking status, age, prescription of a cholinesterase inhibitor/memantine and the use of statins at baseline.

The randomisation service will assign a unique trial identifier to each patient and confirm the study drug pack number to be dispensed by the local pharmacy. The randomisation service will confirm randomisation details by email to the site, local pharmacy and the clinical trials unit (CTU).The unique trial identifier assigned at the time of randomisation will be used throughout the trial for the purposes of patient identification. The online randomisation system will also confirm the study pack numbers to be dispensed at visits 4, 5 and 6.

### Unblinding procedure

As a double-blind placebo-controlled trial, patients, clinicians and the PI will be blinded to each patient’s treatment allocation. Patients will be given a Patient Study Card when they are enrolled in the trial. This will include 24-hour contact details of their treating clinician in the event that the patient needs to contact their clinician. A standardised procedure for emergency unblinding will be available to all participating sites.

Emergency unblinding may be requested on safety grounds, or if the treatment decision for a patient could be influenced by the knowledge of what the patient is taking as part of the trial. If the PI or designated Investigator decides that there is justification to unblind a patient, emergency unblinding will be performed via the online randomisation service. In the event unblinding occurs, the patient may discontinue study drug but will remain on the trial unless they decide to withdraw.

### Study treatment regimen

Patients will be randomised to (1) usual care + amlodipine or (2) usual care + matching placebo.

The usual care for patients will include written information about risk reduction in vascular dementia. This will be based on the most up-to-date evidence and advice. They will then continue to receive the usual care provided by their general practitioner (GP).

Patients will receive 5 mg amlodipine daily or matching placebo for 2 weeks, increasing to 10 mg daily of amlodipine or matching placebo for 50 weeks. The dose will be reduced back to 5 mg or matching placebo daily if intolerable side effects develop. If the dose is reduced to 5 mg there will be no further increase to 10 mg for the remainder of the trial.

Patients should commence study medication (5 mg) the day after the baseline visit and for all subsequent visits patients should take their study medication before attending the visit. Study medication should be administered in the morning. If the study medication is not taken in the morning it can be administered up to 12 hours later. Two doses should not be taken at once.

### Study drug supply

Study drug packs will be packaged and labelled by Victoria Pharmaceuticals (Belfast, UK). Amlodipine 5 mg or placebo will be packaged in containers of 210 tablets and labelled in compliance with the applicable regulatory requirements.

Hospital pharmacies will maintain accurate records of all Investigational Medicinal Product (IMP) received (including date of receipt, batch numbers, expiry date, quantities of drug shipments), dispensed and returned on the Drug Accountability Log. Hospital pharmacies will ensure all study drugs are stored in a secured area under the manufacturer’s recommended storage conditions and held separately from normal hospital stock.

### Study drug compliance

Patients will be asked to store the medication according to the manufacturer’s instructions and should bring all unused medication and empty bottles to each visit. Research staff will perform a count and return any unused medication and empty bottles to the site pharmacy. Patients who have taken 80 % or more of the expected number of tablets will be considered compliant. Non-compliance should be discussed with the PI or designee to determine if appropriate to discontinue medication but continue patient follow up as part of the trial.

### Study drug termination criteria

Prior to the maximum treatment period of 52 weeks (1 year after randomisation), study drug will be discontinued if any of the following conditions are met:

- Patient or informant request termination of study drug
- Patient or informant withdraws consent for the study
- Non-compliance with study drug as determined by the PI (patients who have taken 80 % or more of the expected number of tablets will be considered compliant)
- Side effects remain intolerable following the reduction of the study drug to 5 mg
- Decision by the PI that the study drug should be discontinued on safety grounds

The reason for study drug termination should be recorded and any unused medication returned to pharmacy.

### Study assessments

All patients must be evaluated during the study according to the schedule of assessments (+/- 7 days) outlined in Table 1 and data recorded within the case report form (CRF).

**Table 1:** Schedule of assessments

Visit	1	2			3	4	5	6	7	
Assessment	Screening	Baseline	Week 2	Week 4	Week 6	Week 13	Week 26	Week 39	Week 52	Week 104
Informed consent	x									
Inclusion and exclusion criteria review	x	x								
Patient demographics	x									
Vital signs	x	x			x	x	x	x	x	
Informant demographics	x									
Patient clinical and medical history	x									
Physical examination	x	x							x	
Neurological assessment	x	x							x	
Previous medications	x									
Current medications	x	x			x	x	x	x	x	
Co-morbidities	x									
Biochemistry profile	x	x					x		x	
Full blood count	x	x					x		x	
ECG	x	x					x		x	
MRI	x								x	
Telephone follow-up			x	x						
Cognitive assessments										
VADAS-cog		x			x	x	x	x	x	
CGIC		x				x	x		x	
sMMSE	x	x				x	x		x	
Trail Making B		x				x	x		x	
TICS-M		x					x		x	x
Informant/Patient Questionnaires										
EQ-5D		x				x	x		x	
DAD (informant)		x				x	x		x	
GHQ12 (informant)		x				x	x		x	
EQ-5D (informant)		x				x	x		x	
DEMQOL (informant)		x				x	x		x	
NPI-D (informant)		x				x	x		x	
CSRI (informant)		x				x	x		x	
Service use log		x				x		x		
Advice sheet		x								
Adverse events			x	x	x	x	x	x	x	
Study drug dispensing		x				x	x	x		

*ECG* electrocardiograph, *MRI* magnetic resonance imaging, *VADAS-cog* vascular dementia assessment scale cognitive subscale, *CGIC* clinical global impression of change, *sMMSE* standardised Mini-Mental State Examination, *TIC-M* modified telephone interview for cognitive status, *EQ-5D* EuroQol group 5 dimensions health questionnaire, *DAD* disability assessment in dementia, *GHQ12* 12-item general health questionnaire, *DEMQOL* dementia quality of life, *NPI-D* neuropsychiatric inventory caregiver distress, *CSRI* client service receipt inventory

### MRI assessments

MRI assessments will be completed prior to visit 2 (baseline) to confirm patient eligibility and at visit 7 (52 weeks) as part of the final outcome assessments. Lacunar lesions will be identified and distinguished from mimics such as enlarged perivascular spaces, based on existing criteria. Diffuse white matter abnormalities will be evaluated according to the age-related white matter changes (ARWMC) radiological scale and validated across imaging modalities [30]. The scale grades white matter lesions into punctate, early confluent and confluent, a rating system which has been validated against pathology based on the Fazekas criteria [31]. Lacunar infarcts definitions are based on the STRIVE criteria by Wardlaw et al. [32].

Review of the MRI images will be completed centrally at the AFFECT Imaging Co-ordinating Centre (ICC) overseen by a central team consisting of neurology experts. The central evaluation will be based on a validated scale as reported by Barkhof [33]. During the study a subset of images and screening results will also be provided to the DMEA Committee for the purposes of oversight.

Only once the MRI results are known and all baseline assessments are completed confirming eligibility, should the patient be randomised to the study. Ideally, randomisation should occur within 4 weeks (+/- 7 days) of the screening visit. Patients will be contacted prior to their 52-week visit to schedule the final MRI assessment.

### Current medications

Current medication details will be recorded for each patient at every visit.

If patients are taking cholinesterase inhibitors or memantine, the dose needs to be stable for at least 3 months prior to commencing the study drug. The DMEA Committee will assess patients taking cholinesterase inhibitors or memantine for inclusion in the study.

### Cognitive assessments

All cognitive assessments should be completed face to face by an assessor who will be blinded to the intervention as outlined in Table 1.

At visit 2 (baseline), the cognitive assessments should take place on the same day as the patient is randomised to the study. At visit 7 (52 weeks), the cognitive assessments may take place on the same day as the MRI or pre/post the MRI. Both the MRI and cognitive assessment should be completed within -/+ 7 days of the 52-week visit date. The TICS-M questionnaire is a telephone questionnaire and should be completed the week of the baseline visit but prior to the patient starting the study medication the day after the baseline visit. A telephone follow-up call should also be completed on the week of visits 26, 52 and 104 weeks to complete the modified telephone interview for cognitive status (TICS-M) questionnaire.

### Service use and costs questionnaire

The CSRI will be completed by an assessor in a face-to-face interview with the informant. In addition, a log will be provided to patients at the end of visits 2, 4, and 6. The log is to be used as an optional memory aid to help patients recall service use in the period prior to each assessment point [34].

### Biochemistry profile and full blood count

A blood sample should be taken at visits 1, 2, 5 and 7 and a biochemistry profile and full blood count will be carried out.

### Vital signs

At each visit the patient’s weight, heart rate, sitting (after 10 minutes, second reading) and standing blood pressure (1 minute) should be measured and recorded on the CRF.

An electrocardiogram (ECG) should be carried out at visits 1, 2, 5 and 7. The PI or designee should review and sign off the ECG stating if the results present any non-clinically significant or clinically significant abnormalities. The PI or designee should detail all clinically significant abnormalities.

### Clinical management of patients on the trial

Four weeks following the commencement of patients on study medication (i.e. 2 weeks at 5 mg once daily dose followed by 2 weeks at 10 mg once daily dose), a telephone follow-up call should be completed by each participating site to assess if the patient is experiencing any side effects. It should also be confirmed if the patient has increased the dose of study medication to 10 mg daily. The most common side effects include headache, oedema, flushing, dizziness, ankle swelling, fatigue, nausea, and rash. If the patient reports any intolerable side effects, the PI should be notified who will monitor the patient and decide on the action to be taken.

If there is significant postural hypotension (drop in systolic blood pressure on standing of greater than 20 mmHg), and the patient is taking 10 mg study medication, the dose should be reduced to 5 mg daily. However, if significant postural hypotension persists following the decrease of study medication to 5 mg, the patient will be re-assessed by the PI who will decide whether the study drug is to be stopped.

If the patient’s blood pressure is found to be greater than 160 mmHg systolic or 100 mmHg diastolic, then the patient’s GP should be advised to monitor the patient’s blood pressure to determine if it remains elevated to this level. If the patient’s blood pressure remains elevated, then antihypertensive medication will need to be prescribed. The GP should be advised that any antihypertensive medication, except a CCB can be prescribed.

### Data collection

To ensure accurate, complete and reliable data are collected, training will be provided to site staff in the format of investigator meetings and/or site initiation visits.

All data for an individual patient/informant will be collected by each PI or their delegated nominees and recorded in the electronic database/source document. Patient/informant identification will be through their unique trial identifier allocated at the time of randomisation and initials. Data will be collected and recorded on the CRF and questionnaires by the site research team from the time the patient and their informant are considered for entry into the trial through to their 104-week telephone follow up.

### Adverse events

The PI will record all directly observed adverse events (AEs) and all AEs spontaneously reported by the patient/informant. In addition, the patient/informant will be asked about AEs at each visit following initiation of treatment.

Events associated with the patient’s underlying medical condition should not be reported as AEs. AEs of special interest that should be reported include dizziness and falls. All adverse reactions (ARs), an AE which is related to the administration of the study drug must be reported on the AE form within the CRF. An unexpected adverse reaction (UAR) is an AE which is related to the administration of the study drug and that is unexpected, in that it has not been previously reported in the current SPC. All UARs must be reported on the AE form within the CRF.

All serious adverse events (SAEs), SAEs that are related to the administration of the study drug (SARs) and suspected unexpected serious adverse reactions (SUSARs) will be reported to the CTU within 24 hours of becoming aware of their occurrence. The CTU will inform the Sponsor and regulatory authorities within the required timelines as per the regulatory requirements.

### Sample size

Based on results obtained from the CADASIL study [35], a minimum total sample size of 470 patients will need to be recruited to achieve a small but clinically meaningful standardised effect size of 0.3. Thus 588 patients overall (i.e. 294 patients per treatment group) will be recruited. This is based on a standard deviation of 8.4, and assuming a dropout rate of 20 %, at 90 % power and 5 % level of significance (two-sided).

### Data analysis

The principal comparisons will be performed on an intention-to-treat (ITT) basis. Thus, after randomisation, patients will be analysed according to their allocated treatment group irrespective of what treatment they actually receive. Full follow-up data will be obtained on every patient to allow full ITT analysis, however missing data will be problematic due to withdrawal, loss to follow-up, or non-response response questionnaire items. The results from the trial will be presented as comparative summary statistics (difference in response rates or means) with 95 % confidence intervals. The study results will be reported in accordance with the Consolidated Standards of Reporting Trials (CONSORT) 2010 statements [36]. A fully detailed statistical analysis plan (including plans for any interim analysis, subgroup analysis, and sensitivity analysis) will be prepared before the first interim analysis. The report to the Data Monitoring and Ethics Committee (DMEC) will be prepared approximately 6 months after recruitment has started.

Primary outcome (VADAS-cog) will be analysed using linear mixed-effects models, with repeated measures on outcome measurements at weeks 6, 13, 26, 39 and 52 weeks, adjusting for baseline score, stratification and minimisation variables. Falls will be specifically evaluated as an adverse event of special interest as part of the safety comparison. An interaction between time and randomised group will be fitted to allow estimation of treatment effect at each time point. The distribution of the change from baseline will be formally assessed for evidence of departure from normality. If necessary, data will either be transformed or analysed using a non-parametric equivalent.

The nature and mechanism for the missing outcomes will be investigated, though mixed-effects models implicitly account for data missing at random mechanism. Pre-specified subgroup analysis such as use of statins at baseline will be explored. Sensitivity analyses will be carried out to examine the robustness of the results with different assumptions about departures from randomisation policies, and handling of missing data.

For secondary outcomes, the distributions of the changes in the continuous secondary outcome measures will be formally assessed for evidence of departure from normality. In instances where such changes in outcome are not normally distributed, data will be either transformed and analysed as detailed above or tested using non-parametric equivalents.

The 52-week study data will be published following the collation and analyses of this data set. The 104-week TICS-M data will be published thereafter. The 104-week TICS-M assessment will be an important long-term outcome with valuable information examining the outcomes for patients with SIVD.

### Stopping guidelines

In the light of the DMEC report and other evidence from relevant studies, the DMEC will inform the Trial Steering Committee (TSC) if, in its view, there is proof beyond reasonable doubt that the data indicate the trial should be terminated. It is agreed that recommendations on premature stopping of the trial are more likely to be made on the grounds of safety as there will be no interim analysis carried out for the primary outcome of effectiveness. Therefore, any interim analysis related to the effectiveness of the study will be included in the DMEC report as guidance for benefit-to-risk assessment.

### Economic analysis

Service utilisation patterns, carer inputs and all associated costs will be calculated for each patient, based on data collected from study drug logs and a modified version of the CSRI completed by the informant at baseline, 13, 26 and 52 weeks. Unit costs to reflect long-run marginal opportunity costs will be attached using national figures where available, or calculated anew if necessary.

Cost-effectiveness analyses (CEAs) will be conducted to compare effectiveness and costs over the 52-week period, each from the perspective of (a) the NHS and social services, and (b) society as a whole (including unpaid carer costs).

The primary outcome measure will be the incremental cost of a change in VADAS-cog score from baseline to 12-month follow-up. The secondary outcome measures will be the incremental cost of quality-adjusted life-years (QALY) gained, using utility scores calculated from the DEMQOL-Proxy [37] and from the EQ-5D-5 L with societal weights [38]. Further CEAs will be conducted for other key outcomes: CGIC, sMMSE, DAD, NPI-D, and TICS-M. Cost-effectiveness ratios will be compared with other studies of dementia treatment and (where appropriate) of other treatments, and with National Institute for Health and Clinical Excellence (NICE) thresholds when using QALYs as the effectiveness measure.

Cost comparisons will be made between intervention and control groups, with adjustments probably needed to adjust for non-normality of data (using bootstrap methods or generalized linear modelling). For each hypothesis, relevant perspective and outcome, an incremental cost-effectiveness ratio will be computed and compared with results from other studies where appropriate. Cost-effectiveness acceptability curves will be plotted using bootstrap analyses to address uncertainty and locate the findings of the economic evaluation in their wider decision-making context.

### Trial oversight

The Chief Investigator (CI) will have overall responsibility for the conduct of the study. The Trial Management Group (TMG) will have responsibility for the day-to-day operational management of the trial. The conduct of the trial will be overseen by a Trial Steering Committee (TSC). The TSC comprising investigators, clinicians, trialists and lay members act as the oversight body for the trial on behalf of the Sponsor/Funder. Throughout the trial, the TSC will take responsibility for monitoring and guiding overall progress, scientific standards, operational delivery and protecting the rights and safety of trial patients. An independent Data Monitoring and Ethics Committee (DMEC) will be appointed with responsibility for safeguarding the interests of trial patients, they will monitor the main outcome measures including safety and efficacy and assist and advise the TSC so as to protect the validity and credibility of the trial. A Diagnostic Monitoring and Events Adjudication (DMEA) Committee will also be established to evaluate protocol compliance, safeguard diagnostic accuracy and ensure the inclusion/exclusion criteria are met. This includes a review of MRI scans for patients on cholinesterase inhibitors or memantine and patients who experience disabling stroke/vascular events. In addition, the DMEA Committee will also review a number of randomly selected MRI scans.

## Discussion

Vascular dementia is a very common cause of dementia syndromes. There are no treatments licensed for VaD and there is an urgent need for intervention studies in this condition. It is a heterogeneous condition and the most common subtype is subcortical ischaemic vascular dementia. This study is designed to investigate SIVD in order to maintain a homogeneity within the study group. The agent chosen for intervention is amlodipine, a dihydropyridine calcium channel blocker. There is background information that calcium channel blocking drugs can have useful effects in people with SIVD but that there is a need for improved studies in the area of VaD and SIVD . Amlodipine is a very commonly used agent in this class which has been available for many years for the treatment of hypertension. This study is designed to investigate whether amlodipine can produce benefits compared to placebo in established SIVD. The design of the study, with multiple centres, a double-blind placebo controlled protocol, and central randomization, maximises recruitment opportunities and minimises the risk of selection or allocation bias. It is estimated that the numbers of people with VaD and SIVD will increase globally in the future and the results of this study should inform important treatment decisions.

## Trial status

Recruitment is ongoing.

## Abbreviations

AD, Alzheimer’s disease; ADL, activities of daily living; AE, adverse event; ARWMC, age-related white matter changes; BHSCT, Belfast Health and Social Care Trust; CADASIL, cerebral autosomal dominant arteriopathy with subcortical infarcts and leukoencephalopathy; CCB, calcium channel blocker; CEA, cost-effectiveness analyses; CGIC, clinical global impression of change; CI, Chief Investigator; CONSORT, Consolidated Standards of Reporting Trials; CRF, case report form; CSRI, client service receipt inventory; CT, computed tomography; CTU, clinical trials unit; DAD, disability assessment in dementia; DEMQOL, dementia quality of life; DMEA committee, Diagnostic Monitoring and Events Adjudication committee; DMEC, Data Monitoring and Ethics Committee; DSM-IV, *Diagnostic and Statistical Manual of Mental Disorders, Fourth Edition*; ECG, electrocardiogram; EQ-5D, EuroQol group 5 dimensions health questionnaire; GCP, good clinical practice; GHQ-12, 12-item general health questionnaire; GP, general practitioner; IMP, investigational medicinal product; ISRCTN, International Standard Randomised Controlled Trial Number Register; ITT, intention-to-treat; MRI, magnetic resonance imaging; NICTU, Northern Ireland Clinical Trials Unit; NPI-D, neuropsychiatric inventory caregiver distress; PC-CTU, Primary Care Clinical Trials Unit; PI, Principal Investigator; QALY, quality-adjusted life-years; SAE, serious adverse event; SIVD, subcortical ischaemic vascular dementia; sMMSE, standardised Mini-Mental State Examination; SPC, summary of product characteristics; SUSAR, suspected unexpected serious adverse reaction; TICS-M, modified telephone interview for cognitive status; TMG, Trial Management Group; TSC, Trial Steering Committee; UAR, unexpected adverse reaction; VaD, vascular dementia; VADAS-cog, vascular dementia assessment scale cognitive subscale
